# Developing a computational toolbased on an artificial neural network for predicting and optimizing propolis oil, an important natural product for drug discovery

**DOI:** 10.1371/journal.pone.0283766

**Published:** 2023-05-08

**Authors:** Gayatree Nayak, Akankshya Sahu, Sanat Kumar Bhuyan, Abdul Akbar, Ruchi Bhuyan, Dattatreya Kar, Guru Charan Nayak, Swapnashree Satapathy, Bibhudutta Pattnaik, Ananya Kuanar

**Affiliations:** 1 Centre for Biotechnology, Siksha O Anusandhan University, Kalinga Nagar, Ghatikia, Bhubaneswar, Odisha, India; 2 Institute of Dental Sciences, Siksha ’O’ Anusandhan University, Bhubaneswar, Odisha, India; 3 Department of Biotechnology, Odisha University of Technology & Research, Bhubaneswar, Odisha, India; 4 Department of Medical Research, Health Science, IMS & SUM Hospital, Siksha O Anusandhan University, Bhubaneswar, Odisha, India; 5 Department of Botany, Samanta Chandrasekhar Autonomous College, Puri, India; Jeonbuk National University, REPUBLIC OF KOREA

## Abstract

Propolis is a promising natural product that has been extensively researched and studied for its potential health and medical benefits. The lack of requisite high oil-containing propolis and existing variation in the quality and quantity of essential oil within agro-climatic regions pose a problem in the commercialization of essential oil. As a result, the current study was carried out to optimize and estimate the essential oil yield of propolis. The essential oil data of 62 propolis samples from ten agro-climatic areas of Odisha, as well as an investigation of their soil and environmental parameters, were used to construct an artificial neural network (ANN) based prediction model. The influential predictors were determined using Garson’s algorithm. To understand how the variables interact and to determine the optimum value of each variable for the greatest response, the response surface curves were plotted. The results revealed that the most suited model was multilayer-feed-forward neural networks with an R^2^ value of 0.93. According to the model, altitude was found to have a very strong influence on response, followed by phosphorous & maximum average temperature. This research shows that using an ANN-based prediction model with a response surface methodology technique to estimate oil yield at a new site and maximize propolis oil yield at a specific site by adjusting variable parameters is a viable commercial option. To our knowledge, this is the first report on the development of a model to optimize and estimate the essential oil yield of propolis.

## Introduction

Honeybees generated propolis, a natural resinous mixture made up of plant parts, buds, and exudates. Propolis is now a natural remedy available in a variety of topical forms in many health-food stores. It is used in cosmetics and as a popular alternative medicine for treating a variety of ailments. Cold syndrome (upper respiratory tract infections, common cold, and flu-like disorders) as well as wound healing, burn treatment, acne, herpes simplex, genitals, and neurodermatitis, are treated by propolis. Propolis is also used in mouthwashes and toothpaste to treat gingivitis and stomatitis, as well as to prevent caries. It is found in a variety of cosmetics, as well as health, foods, beverages, Capsules, mouthwash, creams, throat lozenges, powder, and numerous refined items from which the wax has been removed are all commercially available. It has antibacterial, antiviral, and antioxidant effects [1,2]. It is frequently utilized in pharmacology, cosmetics, and human & veterinary medicine. Propolis oil from various geographical origins exhibits varying bioactivity, such as antibacterial activities [3,4] antifungal [5], anticancer [6], and antioxidant [7], and also has a therapeutic effect on anxiety [8]. In the international market, the cost of Bee propolis powder is up to 15000$ per kg. There is currently no credible information on total propolis production around the world, and we continue to lack detailed production in India. Some market study has revealed that global propolis output currently stands at several thousand tonnes. Propolis is in high demand in Japan, Korea, and Taiwan. Propolis collection among Indian beekeepers is non-existent. This is primarily due to a lack of knowledge about the quality of indigenous propolis and its business potential. As a result, scientific research projects to investigate the properties of indigenous propolis are urgently needed. India’s vast floral and crop diversity, as well as its diverse climatic conditions, propolis chemical composition is likely to change across the country. According to numerous studies, the main physiologically active chemicals in propolis are caffeic acids, flavonoids, and phenolic esters. Propolis has a complex chemical composition & its biological effects cannot be directly attributed to these components. The qualitative and quantitative composition of propolis, in our opinion, has a significant impact on their biological activity. Its components and biological activity are influenced by a variety of conditions, including geographical location, time of collection, and plant source [9,10].

As a result, it is critical to investigate the level of heterogeneity in propolis content throughout Odisha’s various agro-climatic areas to better understand the attributes of indigenous propolis for each location. Furthermore, because their production is strongly impacted by environmental conditions, basic chemo typing would not be able to identify the features of indigenous propolis. Various statistical methods are applied to determine the relationship between biochemical content and environmental conditions, Common statistical techniques like correlation and multiple linear regressions (MLR) analyses are only used to identify linear relationships and do not accurately deal with non-linear data [11]. Due to superior prediction performance, artificial neural networks (ANN) are now frequently employed to construct and map non-linear relationships between inputs and outputs. The use of ANN modeling simulates how the human brain works [12]. It is chosen because it can directly learn from situations without analyzing the parameters using statistical techniques [13]. An ANN is divided into three primary sections: the input layer, hidden layers, and the output layer of neurons [14]. The neurons in the input layers take in the input data, and then the incoming data is normalized before being passed to the hidden layer [15]. Each neuron in the layer below calculates a linear combination of the data from the input layer’s neurons, and then adds weight values associated with certain nodes to it.The neurons in the hidden layer combine the linear information from the input layer with a transfer function (a particular non-linear function), which results in the output being the projected model [14]. About the environment and edaphic factors, the ANN model has been used to predict the bioactive content of Podophyllum hexandrum’s podophyllotoxin [16], Hypericum perforatum L’s hyperforin, hypericin, and pseudohypericin [11], and Bacopa monnieri’s bacoside A content [17].

**Fig 1:**
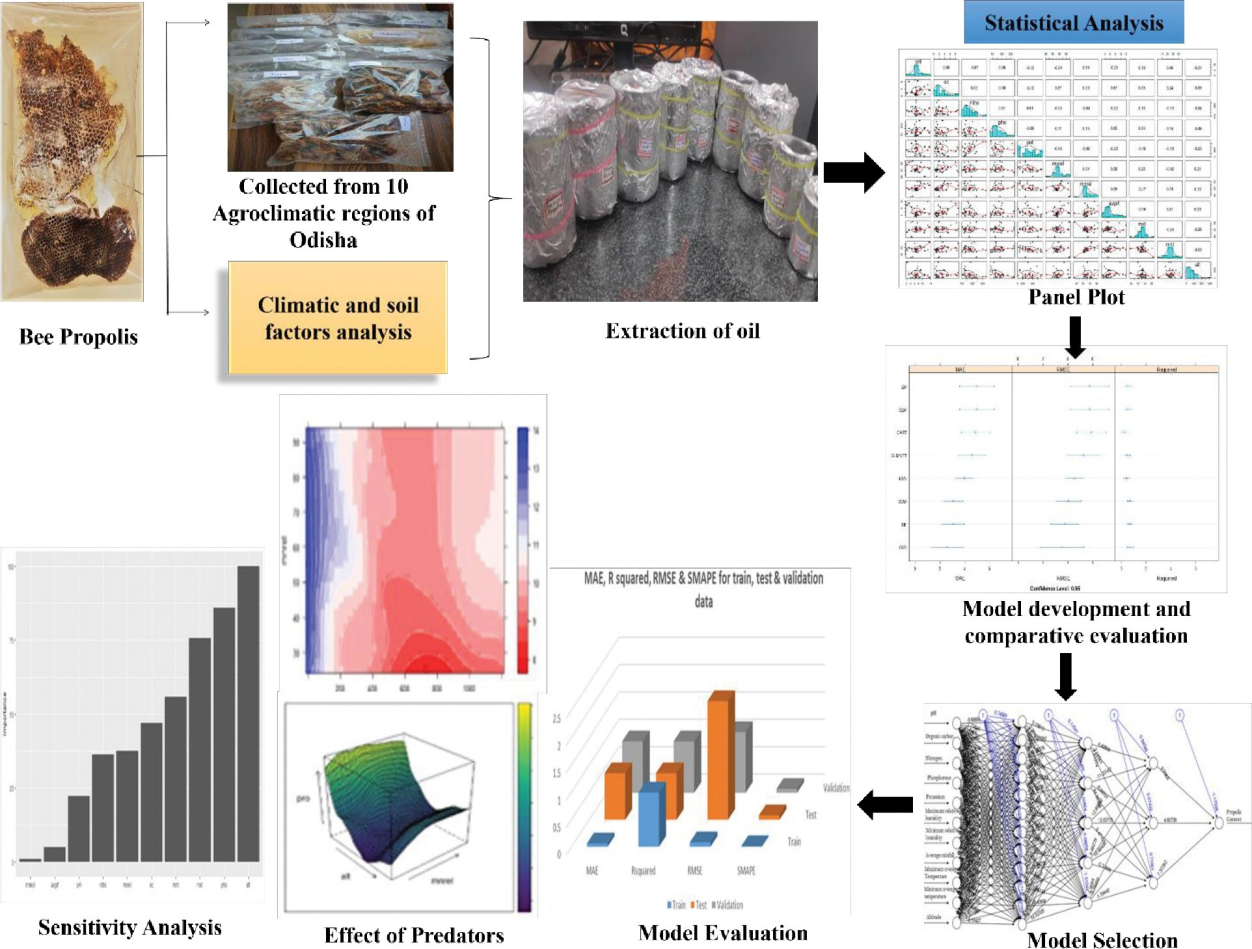
Flow diagram of the study.

In the current work, it will be required to investigate the climatic parameters affecting propolis oil content in various agroclimatic zones in Odisha. So an Artificial Neural Network (ANN) model based on propolis oil content and climatic factors can be developed to predictoptimum yield for the proper regions/sites and to optimize propolis oil yield at a specific region through management of the sensitive and changeable parameters. The flow diagram of this study has been represented in Fig 1.

## Materials and methods

### Propolis sample collection

From late summer June to October 2021, propolis samples were collected from 31 places in Odisha’s ten agro-climatic regions at various altitudes (0.1–1202 m) (Table 1). Propolis samples were obtained in two duplicates from each site. The distance between duplicates was between 2 to 5 meters. To eliminate dust particles, fresh Bee propolis was collected and cleaned with distilled water. Before calculating the propolis oil content, the cleaned propolis was air-dried at room temperature. Each site’s soil samples were collected in duplicates and taken to the lab for investigation of soil nutrients. From June to October 2021, monthly averages of adequately documented data on environmental parameters such as temperature, humidity, and rainfall were obtained from each site (S1 Table).

**Table 1:** Geographic locations and habitats characteristics of propolis.

SL. No.	Agroclimatic Zones	Districts	Accession no.	Latitude	Longitude	Altitude
1.	East & South East Coastal Plain	Jagatsingpur	P1	20.2549° N	86.1706° E	46
			P2	20.2553° N	86.1735° E	43
		Khurda	P3	20.1869° N	86.1737° E	75
			P4	20.1863° N	85.6223° E	181
		Puri	P5	20.1868° N	85.6234° E	0.1
			P6	19.8135° N	85.8312° E	74
		Nayagarh	P7	19.8142° N	85.8318° E	178
			P8	20.1231° N	85.1038° E	14
2.	North Eastern Coastal Plain	Bhadrak	P9	21.0574° N	86.4963° E	23
			P10	21.0570° N	86.4968° E	23.6
		Balasore	P11	21.4934° N	86.9135° E	16
			P12	21.4940° N	86.9140° E	16.3
		Jajpur	P13	20.8341° N	86.3326° E	8
			P14	20.8348° N	86.3330° E	9
3.	North Eastern Ghat	Ganjam	P15	19.3874° N	85.0515° E	3
			P16	19.3870° N	85.0520° E	568
		Gajapati	P17	19.1912° N	84.1857° E	180.5
			P18	19.1918° N	84.1860° E	180.7
		Kandhamal	P19	20.1342° N	84.0167° E	700
			P20	20.1348° N	84.0170° E	591
4.	Mid Central Table Land	Angul	P21	20.8444° N	85.1511° E	876
			P22	20.8450° N	85.1520° E	218.3
		Dhenkanal	P23	20.6505° N	85.5981° E	80
			P24	20.6510° N	85.5988° E	79.6
		Cuttack	P25	20.4625° N	85.8830° E	36
			P26	20.4630° N	85.8840° E	36
5.	Western Central Table Land	Boudh	P27	20.8418° N	84.3200° E	218
			P28	20.8420° N	84.3202° E	221
		Bargarh	P29	21.3470° N	83.6320° E	171
			P30	21.3472° N	83.6322° E	170
		Jharsuguda	P31	21.8554° N	84.0062° E	218
			P32	21.8562° N	84.0065° E	216
6.	Eastern Ghat High Land	Nawarangpur	P33	19.2281° N	82.5470° E	557
			P34	19.2288° N	82.5478° E	553
		Rayagada	P35	19.1712° N	83.4163° E	207
			P36	19.1718° N	83.4169° E	217
		Koraput (East)	P37	18.8561° N	82.7347° E	218
			P38	18.8570° N	82.7355° E	218
7.	North Central Plateau	Mayurbhanj (South)	P39	22.0087° N	86.4187° E	559
			P40	22.0090° N	86.4193° E	564
		Keonjhar (North)	P41	21.6289° N	85.5817° E	596
			P42	21.6287° N	85.5815° E	593
		Mayurbhanj (North)	P43	22.0087° N	86.4187° E	570
			P44	22.0091° N	86.4196° E	596
8.	South Eastern Ghat	Keonjhar (South)	P45	21.6289° N	85.5817° E	193
			P46	21.6285° N	85.5813° E	193
		Koraput (South-East)	P47	18.8561° N	82.7347° E	870
			P48	18.8566° N	82.7354° E	356
		Malkangiri	P49	18.3436° N	81.8825° E	178
			P50	18.3441° N	81.8821° E	170
9.	North Western Plateau	Sundargarh	P51	22.1240° N	84.0432° E	233
			P52	22.1248° N	84.0437° E	231
		Deogarh	P53	21.5383° N	84.7289° E	254
			P54	21.5388° N	84.7293° E	253
		Sambalpur	P55	21.4669° N	83.9812° E	135
			P56	21.4673° N	83.9818° E	252
10.	Western Undulating Zone	Kalahandi	P57	19.9137° N	83.1649° E	355
			P58	19.9141° N	83.1653° E	352
		Bolangir	P59	20.7011° N	83.4846° E	383
			P60	20.7017° N	83.4848° E	556
		Nuapada	P61	20.8060° N	82.5361° E	1200
			P62	20.8068° N	82.5368° E	1202

### Extraction and quantificationof propolis oil

The propolis oil was extracted by combining 10g of cleaned propolis with 200 ml of any refined food quality oil (e.g. coconut oil, sunflower oil, etc.) or 100 g of butter. After that, the components were gently heated in a water bath (no more than 50°C) for around 10 minutes while being constantly stirred. The oil was then purified and stored in the dark in well-sealed containers for future use.

### Quantitative analysis of soil

The samples of soil were taken from each sampling site in Odisha’s ten agro-climatic areas. The soil data from different Agro-climatic regions of Odisha were represented in S2 Table. Approximately 200 g of soil was collected and sieved through a 2 mm mesh. For nutrient analysis, the fine soil particles were extracted, and the pH of a 1:2 soil: water suspension was evaluated using the Systronics pH meter after 30 minutes of equilibration with occasional stirring (Model MKVI). The Walkley and Black wet digestion methodwereused to assess the organic carbon content as described in soil chemical analysis, [18] whereas the total nitrogen was determined using alkaline KMnO4 as described in soil chemical analysis [19]. In an 800ml Kjeldahl flask, 20 g of soil sample was added to 100 ml of 0.32 percent KMnO4 solution, followed by 2.5 percent NaOH solution and distilled water. In a 250 mL conical flask containing 20 mL boric acid (2%) and mixed indicator, distillation was continued and collected in a receiver tube. The distillate was titrated in a burette against 0.02 N H2SO4 to a pink color endpoint and the amount of accessible nitrogen was determined.

Brays No-1 techniques were used to estimate total phosphorus in soil samples. The 2 grams of soil were extracted using 40 ml of Bray’s-1 solution (0.025 N HCl and 0.03 NH4F) and mechanically agitated for 5 minutes before filtering through the Whatman filter paper. A 0.5 ml aliquot was transferred to a flask with a capacity of 25 mL. The volume was increased to 25 ml by adding distilled water and 5 mL ammonium molybdate solution. The volume was made up to the mark with diluted SnCl2 (0.5 ml was diluted to 66 ml). A spectrophotometer (Model: Systronics 166) was used to measure phosphorus content at 660 nm. The concentration was determined using a standard graph made from various phosphorus concentrations. 5 g of soil samples were placed in a 100 ml conical flask with 25 ml of 1 N NH4OAc solution, and the potassium content of the soil was measured. The filtrate was then shaken for 5 minutes with a mechanical shaker, and the potassium content was evaluated using a flame photometer (Model: Sistronics128).

### Statistical analysis

#### Data exploration

All computational work (model development, plot generation, etc.) was performed by using R [20] & Microsoft Excel 2013. The data set consists of 12 features & 62 instances. Out of the features, 11 are predictors. The predictors are soil pH (pH), organic carbon (oc), nitrogen (nitro), the phosphorous (pho), potassium (pot) content the of soil, maximum relative humidity (mxrel), minimum relative humidity (mnrel), average rainfall (avgrf), maximum average temperature (mxt), minimum average temperature (mnt) and altitude (alt). Propolis content is the response. Standard deviations for all features are calculated by using the mlbench [21] library. The formula is provided below.

$$Standard\;deviation=\sqrt{\frac{{\left(x-\bar{x}\right)}^{2}}{n-1}}$$ Eq 1

where *x*&$\bar{x}$ are value of each observation & mean of all observations respectively.

Pearson’s correlation coefficient between features and data distribution in each feature was evaluated by using the package psych [22]. The correlation values were provided in the plot in the form of numeric values as well as a correlation ellipse.

$$The\;Pearson’s\;Correlation\;coefficient\;(r)=\frac{\sum \left\{(x-\bar{x})(y-\bar{y})\right\}}{\sqrt{\sum {\left(x-\bar{x}\right)}^{2}\sum {\left(y-\bar{y}\right)}^{2}}}$$ Eq 2

where *x* &*y* are the values of the two variables; $\bar{x}\&\bar{y}$ are the respective means.

A panel plot was generated to find out the data disribution, and multicolinearity among the variables. For this purpose, a psych package was used.

#### Data splitting

The dataset is divided into three sets, train, test & validation with 70%, 20% & 10% of data. A train set was used to develop the model by training. A test set was used to evaluate the model. Finally, the model was validated by using a validation set.

#### InitialModel development

A comparative modeling approach was applied to identify the best-performing model for the dataset. Several linear algorithms viz. linear regression model (LM), generalized linear regression (GLM), Penalized linear regression model (GLMNET); nonlinear algorithms viz. K-nearest neighbors (KNN), support vector machine (SVM), artificialneural network (ANN) &tree-based models viz. classification & regression tree (CART) &random forest (RF) were developed & evaluated. The resampling method used wascross-validation. The data waspreprocessed withmin-max normalization. Coefficient of determination (R—squared), root mean square error (RMSE), andmean absoluteerror (MAE)were used to evaluatethe models. For this purpose, Caret (classification & regression training) package was used.

#### Model evaluation & selection

Out of theabovemodels,the model which had the highest R -squaredvalue & lowest root mean square error (RMSE), mean absolute error (MAE) & coefficient of determination (R-squared) values of model for the train, test & validation data set were calculated using the following equations.


$$RMSE==\sqrt{\frac{\sum {\left(\hat{y}-y\right)}^{2}}{n}}$$ Eq 3

$$MAE==\frac{\sum \left(y-\hat{y}\right)}{n}$$ Eq 4

$$R\;squared=\frac{\sum {\left(\hat{y}-\bar{y}\right)}^{2}}{\sum {\left(y-\bar{y}\right)}^{2}}$$ Eq 5

The caret (classification & regression training) package was used to develop the final model. Data was scaled using minimum-maximum normalization. The train set was resampled with cross-validation during training. A grid-tuning approach was applied to find an optimum number of layers & nodes in each layer. A resilient backpropagation algorithm with weight bracketing was used for training. The Logistic function was selected as the activation function. The learning rate was kept at 0.4. The Sum of squared errors (SSE) was used for the calculation of errors.

$$SSE=\sum {\left(y-\hat{y}\right)}^{2}$$ Eq 6

where *y* & $\hat{y}$ are the actual response & predicted response respectively.

#### Model tuning

The ANN model was further fine-tuned to improve its prediction performance. The data were preprocessed using the minimum-maximum normalization method. Several hyperparameters are tuned to develop the final model. Those were the number of hidden layers, the number of nodes in each layer, the number of folds in resampling, the activation function & the learning rate. The input data is resampled with cross-validation. Agrid-tuning approach was applied to find an optimum number of layers & nodes in each layer.

The end model was selected based on several metrics *viz*. symmetricmean absolute percentage error (SMAPE), Nash—Sutcliffe efficiencycoefficient (NSE) along with RMSE, MAE & R squared values. The model was also analyzed with regression & slope-intercept tests as proposed by Rocabruno-Valdes et al. (2019) [23].

SMAPE iscalculated byusing the following formula.


SMAPE=(1/n)*Σ(|predicted–actual|/((|actual|+|predicted|)/2)*100

Where predicted & actual are the predicted values by the final model & actual values respectively.

Several authors have used SMAPE to evaluate & improve their developed models by using SMAPE [24,25]. SMAPE was calculated with the help of Microsoft Excel 2013.

Nash—Sutcliffe efficiency coefficient (NSE) was used for the final selection of the model. When the NSE value is 1, the predicted value and actual values are the same. If the NSE value is zero, the predicted values match the mean of theobserved values. When the value is below less than 0, the model is not significant [26].

#### Effect of predictors

Partial dependence plots (PDP) were generated to investigate the interaction of predictors with the response. These plots were generated by using the PDP library. PDP plots are used to interpret the output of complex machine-learning models [27]. The use of linear plots is ineffective for explaining the complex relationships between various variables and the response. In this study, single-variable &multiple-variable PDPs are generated. Smoothing is applied by using locally weighted regression (LOESS) in the case of single variable PDPs. It has popularity in the smoothing of scatter plots [28]. LOESS can perform well even if the response is a nonlinear function of the predictor [29]. The relationships of the response variable with two predictors are represented by two-dimensional contour & three-dimensional PDPs.

#### Sensitivity analysis

It’s critical to figure out what the most crucial factors are that influence propolis oil yield. As a result, the influential predictors were determined using Garson’s algorithm. The relative importance of a certain predictor is obtained by finding the link strengths between the node [30]. The variable importance was evaluated by using Neural Network Tools [31] library.

## Results

### Data exploration

**Fig 2:**
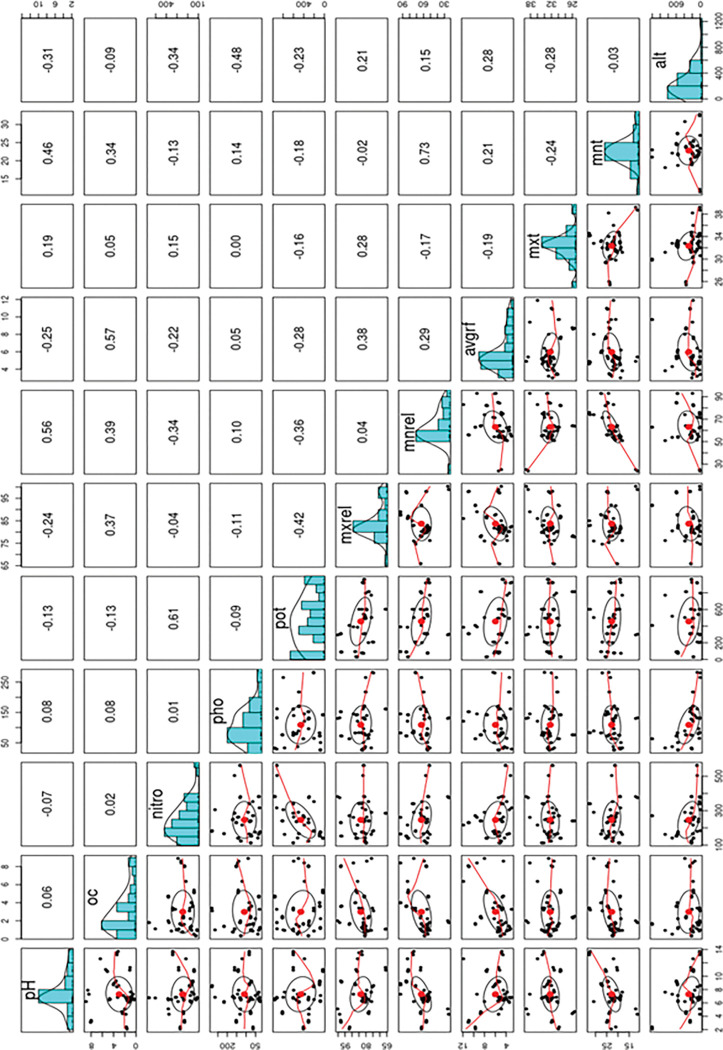
Panel plot to investigate the interaction among predictors.

The panel plot in Fig 2 shows four different representations of the data set through scatter plots, ellipse plots, histograms & Pearson’s correlation coefficients. The scatterplots with trend lines represent the linear relationship between two variables. The correlation ellipse shows the correlation strength. The higher the stretch, the higher the correlation coefficient. The histogram plots show the data distribution for each variable.Pearson’s correlation coefficients are represented in numeric values. The correlation coefficient values range from-0.48 (between soil phosphorous content to altitude) to +0.73 (between minimum relative humidity & minimum average temperature). The positive& negative signs of the correlation coefficients represent negative & positive correlation respectively. No correlation was observed between soil phosphorous content & maximum average temperature. A negligible correlation was observed between pH & organic carbon content (correlation coefficient = 0.06), pH & soil nitrogen content (correlation coefficient = -0.07), etc. A weak correlation was observed betweenphosphorous & minimum relative humidity (correlation coefficient = 0.1), organic carbon & minimum average temperature (correlation coefficient = 0.34), etc.A strong correlation is observed between minimum relative humidity& minimum average temperature (correlation coefficient = 0.73).

From the panel plot (Fig 2), it is evident that some of the predictors *viz*. pH, maximum relative humidity (mxrel), minimum relative humidity (mnrel), maximum average temperature (mxt) & minimum average temperature (mnt) are normally distributed. Some of the predictors have a positively skewed distribution. organic carbon (oc), soil nitrogen content (nitro), phosphorous (pho), average rainfall (avgrf), and altitude (alt) show positive skewness. Soil potassium content (pot) showed platykurtik distribution.Standard deviations of all variables are provided in Table 2.

**Table 2:** Standard deviation of predictors.

Factors	Standard Deviation
pH	2.63
Organic carbon	2.37
Nitrogen	106.78
Phosphorous	62.95
Potassium	289.62
Maximum relative humidity	7.57
Minimum relative humidity	14.2
Average rainfall	2.19
Maximum average temperature	2.56
Minimum average temperature	3.99
Altitude	272.61
Organic carbon	2.37

### Model selection

**Fig 3:**
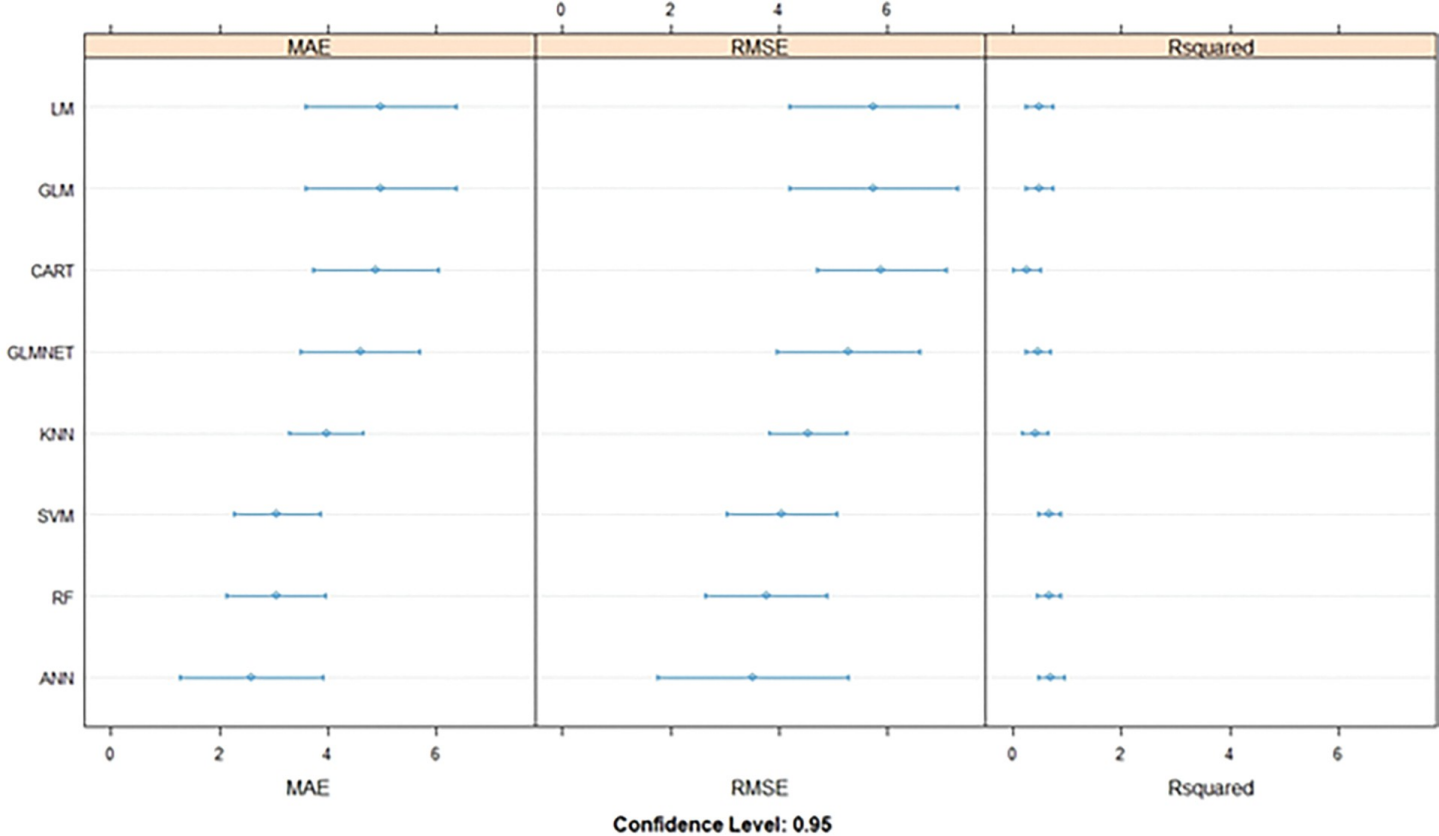
Comparative evaluation of models based on MAE, RMSE & R-squared values.

**Fig 4:**
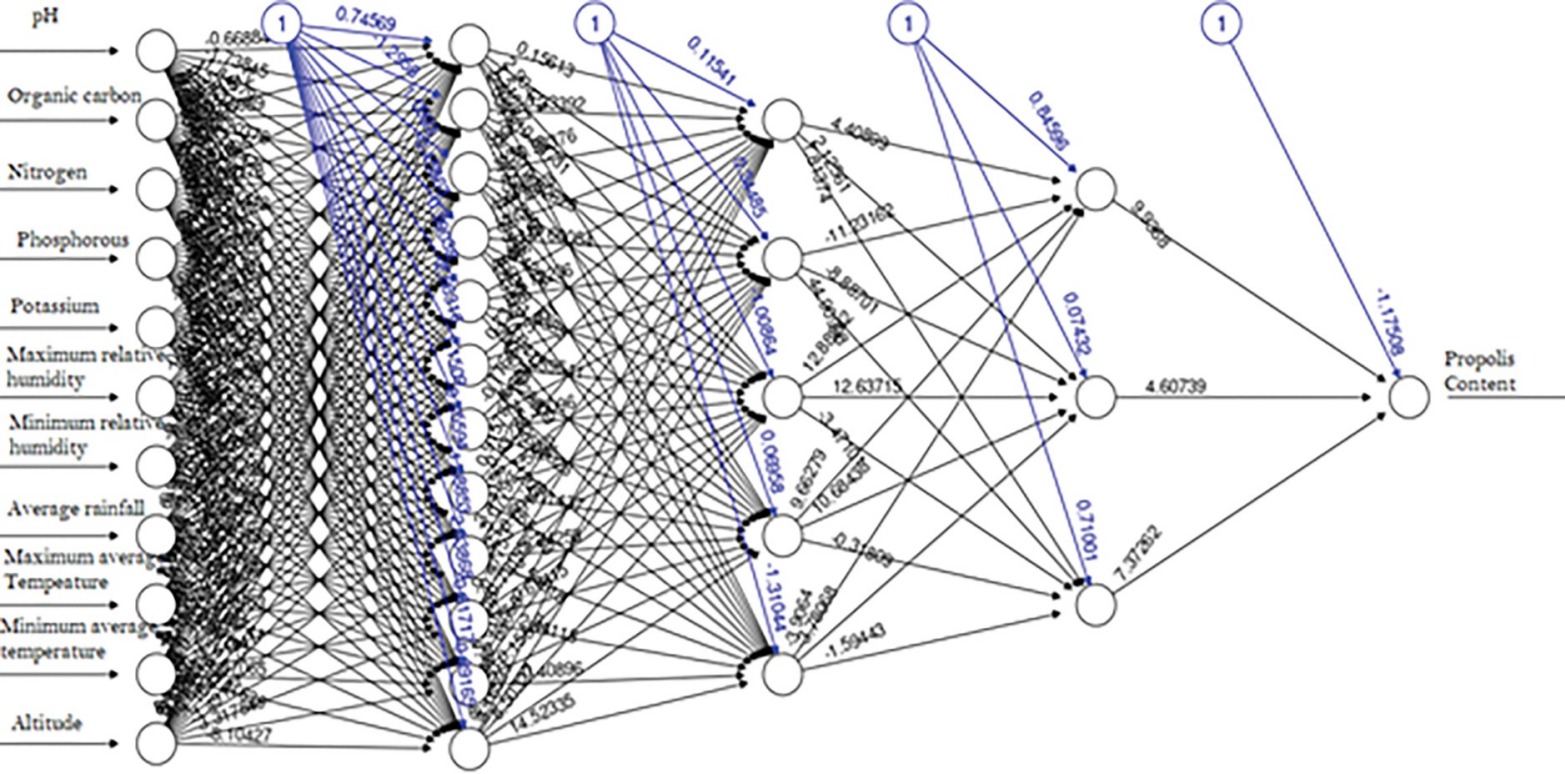
The selected Resilient back propagation ANN model with weight bracketing with three hidden layers, bias & connection strengths.

Eight different modelswere developed. The MAE,RMSE & R-squaredof the developed modelswere provided in Table 3 & Fig 3. Outof these models, the artificialneural network (ANN)model was found to have a better performancemeasure as compared to other algorithms. Thefinal model had five layers withone input layer, one output layer, andthreehidden layers. The inputand output layers had 11and 1 neurons or nodes respectively.Thefirst hidden layer had 12 nodes;the second &third hidden layershad 5& 3nodes respectively. The model along with layers, nodes & weights was provided in Fig 4.

**Table 3:** Comparative evaluation of models based on MAE, RMSE & R-squared values.

Model	MAE	RMSE	R-squared
**LM**	4.9	5.7	0.48
**GLM**	4.9	5.7	0.48
**CART**	4.9	5.9	0.26
**GLMNET**	4.6	5.3	0.46
**KNN**	3.9	4.5	0.41
**SVM**	3.1	4.0	0.66
**RF**	3.0	3.8	0.66
**ANN**	2.6	3.5	0.70

**Fig 5:**
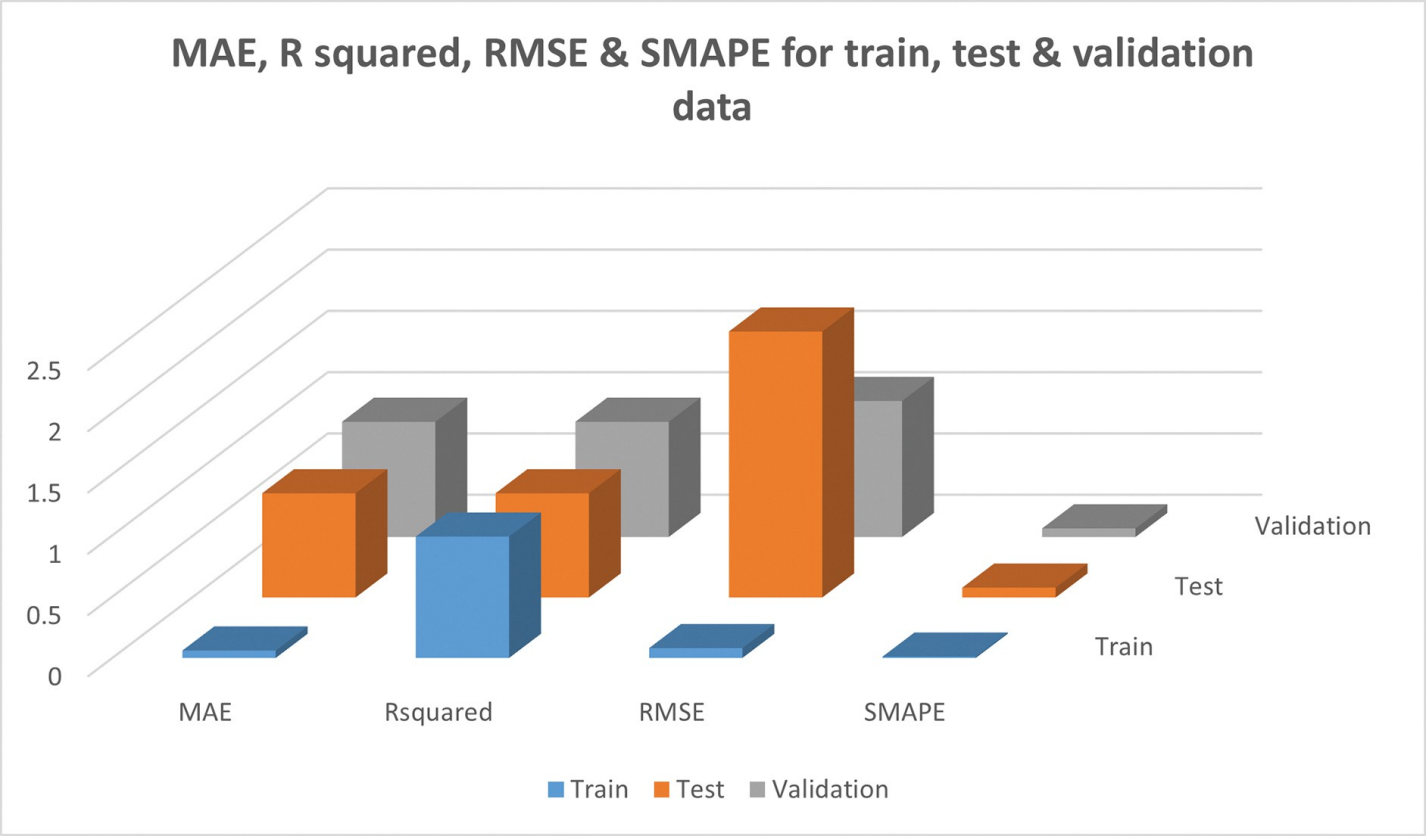
Mean absolute error (MAE), R squared, root mean squared error (RMSE), symmetric mean absolute percentage error (SMAPE) for train, test & validation data.

The final model was selected based on the previously discussed metrics i.e. RMSE, MAE & R- squared (Table 3 & Fig 3).Thefinal model’s performancewas further evaluatedwith SMAPEscores,provided in Table 4 & Fig 5. The NSE values fortrain,testand validationsets are 0.99, 0.83 & 0.92 respectively.

**Table 4:** MAE, R-squared, RMSE & SMAPE of the final model.

	MAE	R-squared	RMSE	SMAPE
Train	0.06	0.99	0.08	0.01
Test	0.85	0.85	2.17	0.08
Validation	0.94	0.94	1.11	0.07

Theregression statistics are presentedin Table 5. The table showstheR-squared, adjusted R-squared values,and standard error of regressionfor train,test, and validation sets.

**Table 5:** Regression test.

Data set	R-squared	Adjusted R-squared	*Std*. *Error of regression*
Train	0.99	0.99	0.07
Test	0.85	0.84	2.02
Validation	0.94	0.93	1.29

Theresults of slopeand interceptanalysisfor train, test,and validationsetsare providedin Table 6A, 6B and 6C respectively.

**Table 6:** 

**Table 6a Slope& Intercept Test for train set:**						
* *	*Coefficients*	*Standard Error*	*t Stat*	*P-value*	*Lower 95%*	*Upper 95%*
Intercept	-0.04	0.02	-1.81	0.08	-0.09	0.00
Slope	1.0	0.00	523.44	4.79E-80	0.99	1.0
**Table 6b Slope & Intercept Test for test set:**						
* *	*Coefficients*	*Standard Error*	*t Stat*	*P-value*	*Lower 95%*	*Upper 95%*
Intercept	1.07	1.31	0.81	0.43	-1.82	3.95
Slope	0.84	0.11	7.9	7.38E-06	0.61	1.07
**Table 6c Slope & Intercept Test for validation set:**						
* *	*Coefficients*	*Standard Error*	*t Stat*	*P-value*	*Lower 95%*	*Upper 95%*
Intercept	-1.12	1.81	-0.62	0.57	-6.15	3.9
Slope	1.07	0.13	8.08	0.00	0.7	1.43

**Fig 6:**
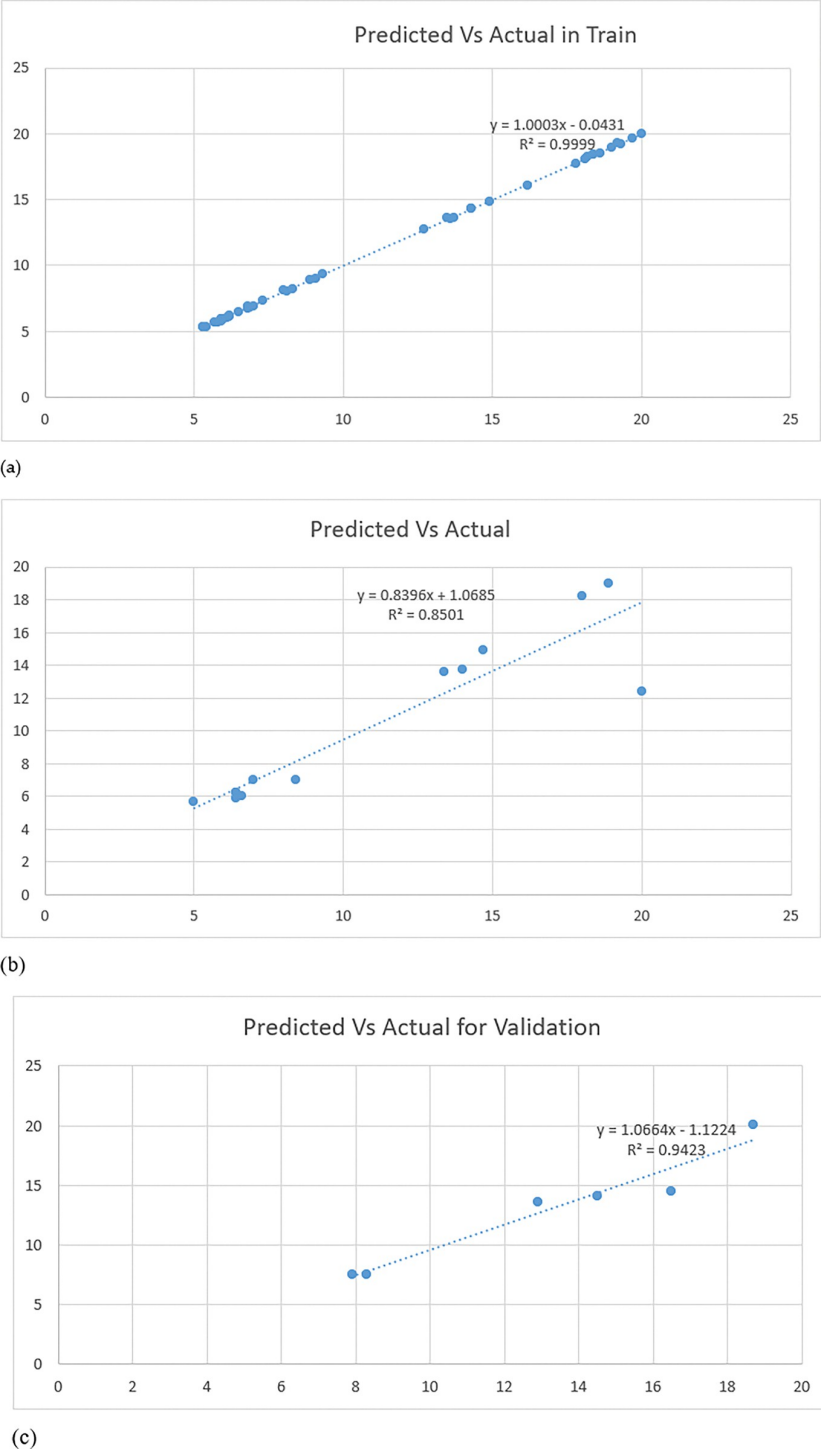
Scatter plot showing the experimental and predicted value of propolis. (a) train; (b) test and (c) validation data.

The predictions & the actual responses for the train set were provided in Table 7 & Fig 6A. The RMSE, MAE & R-squared values of the model for the train set were 0.08, 0.06 & 0.99 respectively. The model was evaluated using the test data set once it had been trained. The RMSE, MAE &R squared values were 2.17, 0.85 & 0.85 respectively.Further, the model was validated with a validation set. The model also performed well with RMSE, MAE & R squared values of 1.11, 0.94 & 0.94 respectively.The predictions & the actual responses for the test &validation sets were provided in Tables 8 & 9 and Fig 6B & 6C respectively.To the best of our knowledge, this is the first study to provide insight into propolis oil content prediction.

**Table 7:** Predicted & actual propolis content for a train set.

SL. No.	Agroclimatic Zones	Districts	Accession no.	Experimental Propolis yield	Predicted Propolis yield	Absolute =
				(X_1_)	(X_2_)	|X_1_-X_2_|
1.	East & South East Coastal Plain	Jagatsingpur	P1	7	6.9	0.1
			P2	6.8	6.9	0.1
		Khurda	P3	7.3	7.3	0
			P6	6.8	6.7	0.1
		Nayagarh	P7	6.1	6	0.1
			P8	6.2	6.2	0
2.	North Eastern Coastal Plain	Bhadrak	P9	5.3	5.3	0
			P10	5.9	5.9	0
		Balasore	P11	6	5.9	0.1
			P12	5.7	5.7	0
		Jajpur	P13	5.9	5.8	0.1
			P16	20	20	0
		Gajapati	P17	19	18.9	0.1
			P18	19.3	19.2	0.1
		Kandhamal	P19	18.2	18.2	0
			P20	18.1	18.1	0
		Dhenkanal	P23	18.4	18.4	0
			P24	18.6	18.5	0.1
			P26	17.8	17.7	0.1
3.	Western Central Table Land	Boudh	P27	13.5	13.6	0.1
		Bargarh	P29	12.7	12.7	0
			P30	13.6	13.7	0.1
		Jharsuguda	P31	14.9	14.8	0.1
4.	Eastern Ghat High Land	Nawarangpur	P33	8.1	8	0.1
			P34	8	8.1	0.1
		Rayagada	P35	8.9	8.9	0
			P36	8.3	8.2	0.1
		Koraput (East)	P37	9.3	9.3	0
			P38	9.1	9	0.1
5.	North Central Plateau	Mayurbhanj (South)	P39	5.8	5.7	0.1
			P40	5.4	5.3	0.1
			P42	6.2	6.1	0.1
			P44	6.9	6.8	0.1
			P46	16.2	16.1	0.1
		Koraput (South-East)	P47	14.3	14.3	0
		Malkangiri	P49	13.6	13.5	0.1
			P50	14.3	14.3	0
		Deogarh	P53	19.7	19.7	0
			P54	19.2	19.3	0.1
			P56	18.4	18.4	0
6.	Western Undulating Zone	Kalahandi	P57	6.5	6.5	0
		Bolangir	P59	5.9	5.9	0
			P60	5.3	5.3	0

**Table 8:** Predicted & actual propolis content for the test set.

SL. No.	Agroclimatic Zones	Districts	Accession no.	Experimental Propolis yield	Predicted Propolis yield	Absolute =
				(X_1_)	(X_2_)	|X_1_-X_2_|
1.	East & South East Coastal Plain	Khurda	P4	8	8.4	0.4
		Puri	P5	6.2	6.4	0.2
2.	North Eastern Ghat	Ganjam	P15	13.6	13.4	0.2
3.	Mid Central Table Land	Angul	P21	14.9	14.7	0.2
		Cuttack	P25	13.7	14	0.3
4.	North Central Plateau	Keonjhar (North)	P41	5.7	5	0.7
		Mayurbhanj (North)	P43	7	7	0
5.	South Eastern Ghat	Keonjhar (South)	P45	12.4	13	0.6
6.	North Western Plateau	Sundargarh	P51	19	18.9	0.1
		Sambalpur	P55	18.2	18	0.2
7.	Western Undulating Zone	Kalahandi	P58	6	6.6	0.6
		Bolangir	P61	6.2	6.4	0.2
		Nuapada	P62	5.9	6.4	0.5

**Table 9:** Predicted & actual propolis content for the validation set.

SL. No.	Agroclimatic Zones	Districts	Accession no.	Experimental Propolis yield	Predicted Propolis yield	Absolute =
				(X_1_)	(X_2_)	|X_1_-X_2_|
1.	North Eastern Coastal Plain	Jajpur	P14	8.3	7.5	0.8
2.	Mid Central Table Land	Angul	P22	19.7	20.1	0.4
3.	Western Central Table Land	Boudh	P28	14.5	14.1	0.4
		Jharsuguda	P32	12.9	13.6	0.7
4.	South Eastern Ghat	Koraput (South-East)	P48	16.5	15.9	0.6
5.	North Western Plateau	Sundargarh	P52	19.7	20.1	0.4

As measured by statistical criteria such as coefficient of determination (R^2^) and root mean square error (RMSE) values, the ANN model built in this study demonstrated strong predictive potential for propolis oil content. The stronger the ANN model is, the closer the R^2^ value is to 1 and the lower the RMSE value. As a result, it’s possible to conclude that the model’s propolis oil content prediction is quite accurate.

### Identifying significant predictor

**Fig 7:**
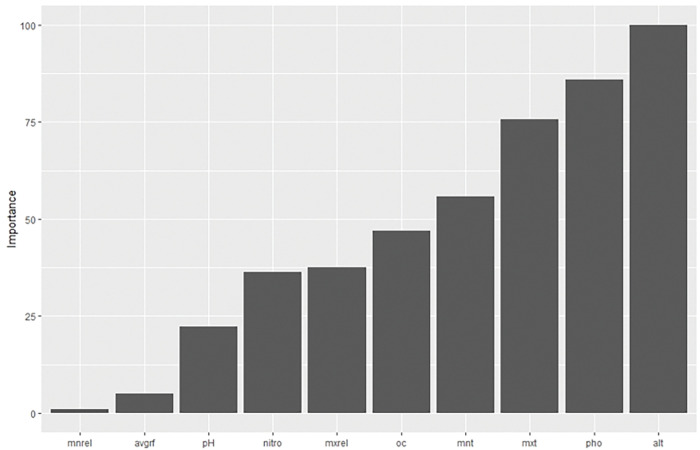
Relative importance of factors.

According to the model, altitude was found to have a very strong influence on response, followed by phosphorous & maximum average temperature. Minimum relative humidity was found to have the least effect on propolis content. The relative importance of all variables on importance is provided in Fig 7 Garson’s method of importance calculation removes the predictor which is insignificant,and soil potassium content was not shown. According to the model, altitude was found to have a very strong influence on response, followed by phosphorous & maximum average temperature. Garson’s algorithm was used to determine the influential predictors.

### Effect of individual predictors on propolis content

**Fig 8:**
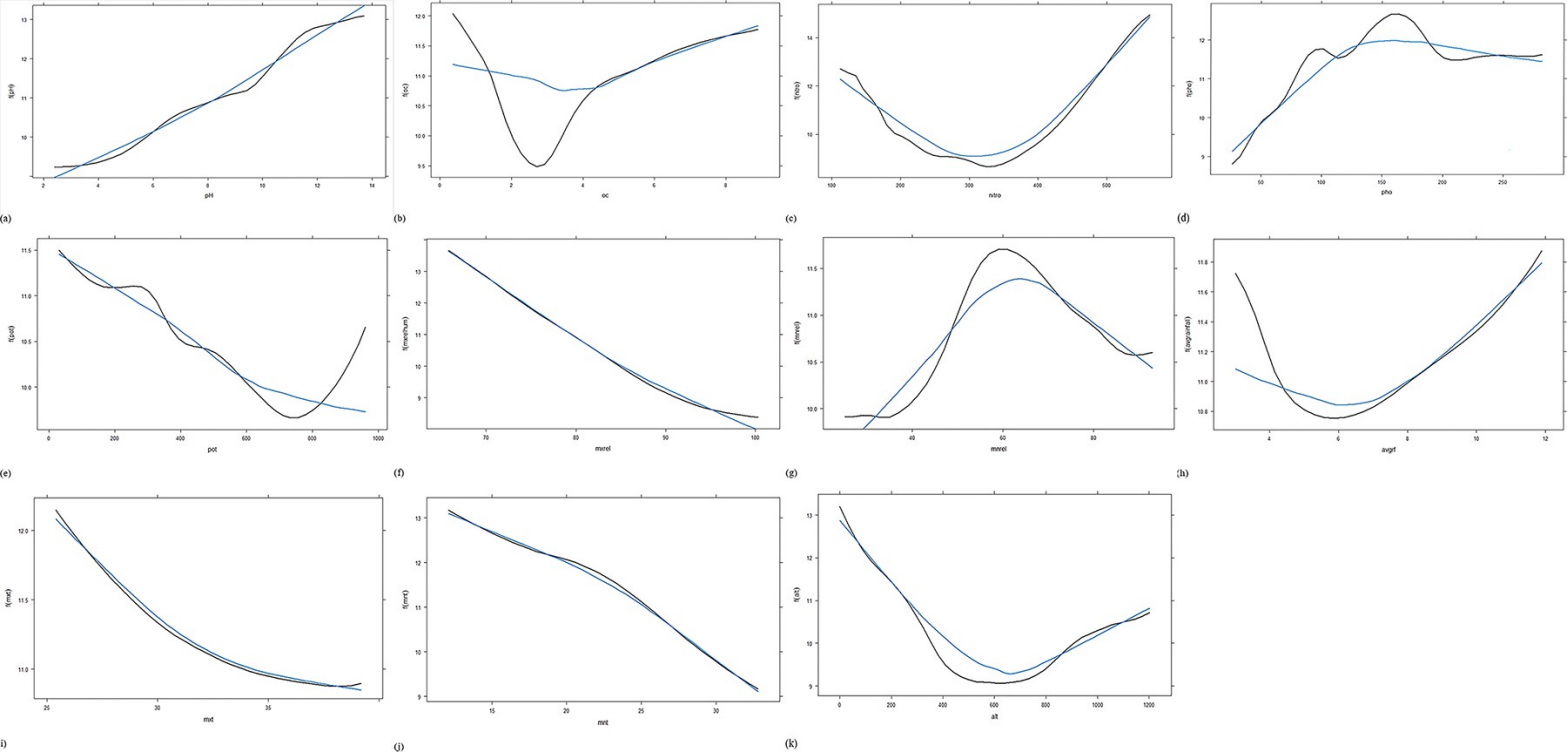
(a)Partial dependence plot of response with respect to pH; (b) Partial dependence plot of response with respect to organic carbon; (c) Partial dependence plot of response with respect to nitrogen; (d) Partial dependence plot of response in terms of phosphorous; (e) Partial dependence plot of response in terms of potassium; (f) Partial dependence plot of response in terms of maximum relative humidity; (g) Partial dependence plot of response in terms of minimum relative humidity; (h) Partial dependence plot of response in terms of average rainfall; (i) Partial dependence plot of response in terms of maximum average temperature; (j) Partial dependence plot of response in terms of minimum average temperature; (k) Partial dependence plot of response in terms of altitude.

Single variable PDPs are generated for all predictors & provided in Fig 8A–8K. The multicollinearity among the features of the data set is described. Most of the correlation among variables is weak to moderate. Minimum average temperature & minimum average relative humidity have a strong correlation. The variation of propolis content with altitude (the most significant predictor) was provided,with a lower value of altitude, propolis content was found to be higher. From the figure, it is evident that the response value gradually decreases with an increase in altitude. The propolis content reaches a minimum between altitudes of 400m to 800m. After 800m., propolis content increases, but at a lower rate. Similarly, the optimum phosphorous content was found to be between 125 & 200 kg/ha (Fig 8D). Propolis content was found to be higher when both maximum & minimum average temperature values are lower. With the increase in temperature, propolis content decreases gradually (Fig 8I and 8J). Propolis content was lower when the organic carbon content of the soil was between 1.5 to 4 kg/ha. Maximum relative humidity was negatively correlated with propolis content (Fig 8F). Propolis content was maximum when the minimum relative humidity is approximately 60 (Fig 8G). The effect of nitrogen, pH, average rainfall & potassium content of the soil was represented in Fig 8A, 8C, 8H and 8E respectively. The variable importance of input parameters on oil yield (output) is shown in Fig 7.

### Mutual effect of two predictors on response

**Fig 9:**
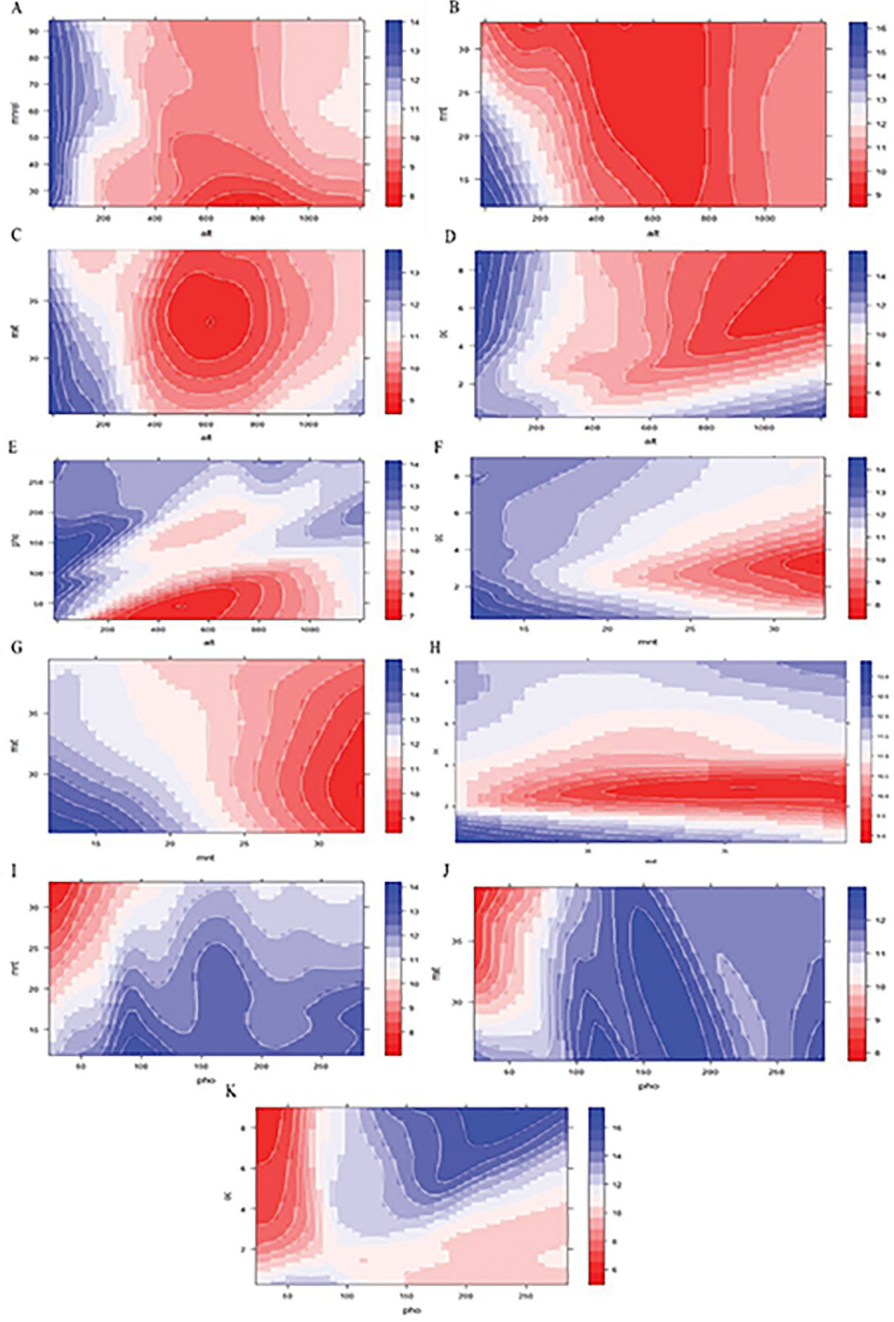
A. Contour plot of altitude, min. rel. humidity & propolis content. B. Contour plot of altitude, min. temperature & propolis content. C. Contour plot of altitude, max. temperature & propolis content. D. Contour plot of altitude, organic carbon & propolis content. E. Contour plot of altitude, phosphorus & propolis content. F. Contour plot of min. temperature, organic carbon & propolis content. G. Contour plot of min. temperature, max. temperature & propolis content. H. Contour plot of max. temperature, organic carbon & propolis content. I. Contour plot of phosphorus, min. temperature & propolis content. J. Contour plot of phosphorus, max. temperature & propolis content. K. Contour plot of phosphorus, organic carbon & propolis content.

**Fig 10:**
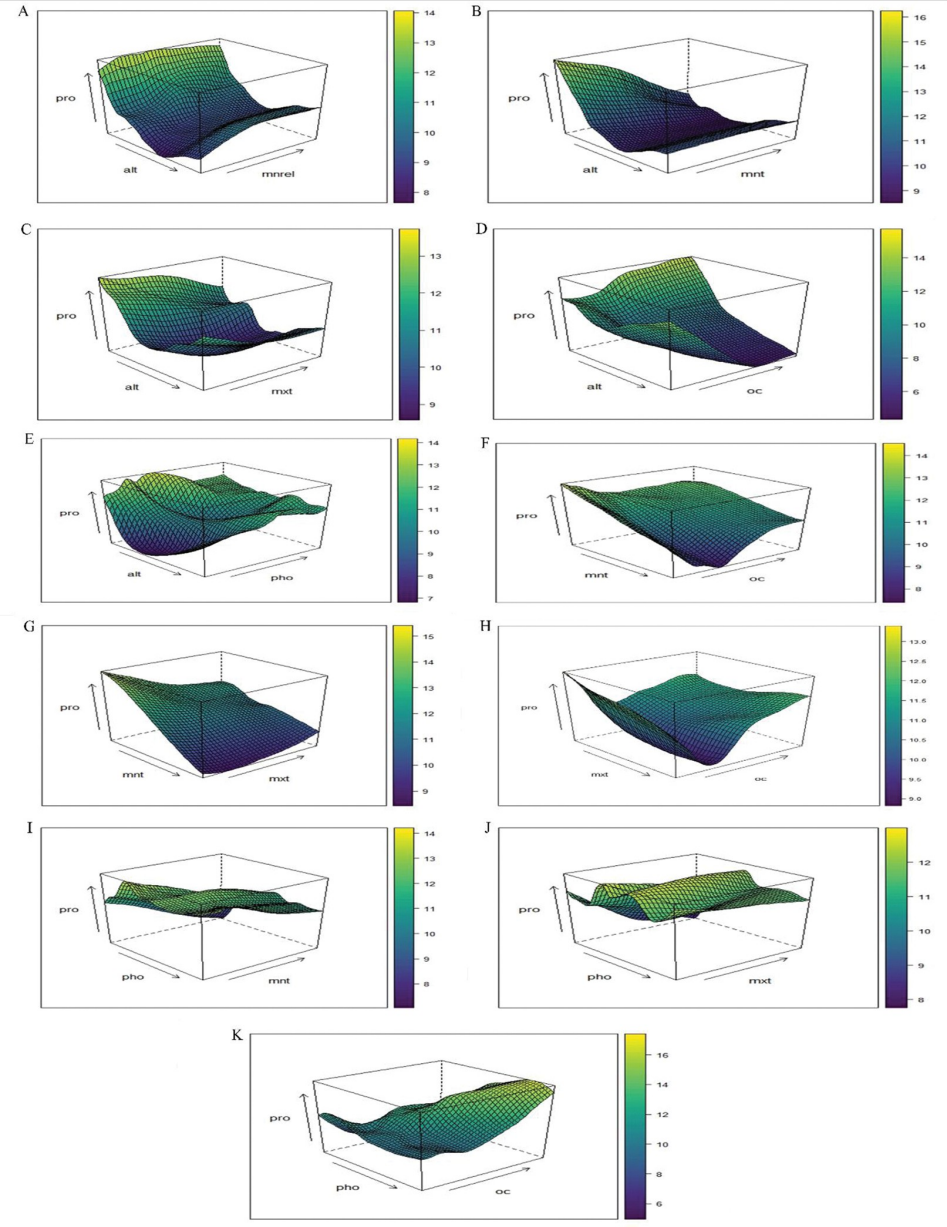
A. 3D PDP of altitude, min. rel. humidity & propolis content. B. 3D PDP of altitude, min. temperature & propolis content. C. 3D PDP of altitude, max. temperature & propolis content. D. 3D PDP of altitude, organic carbon & propolis content. E. 3D PDP of altitude, phosphorus & propolis content. F. 3D PDP of min. temperature, organic carbon &propolis content. G. 3D PDP of min. temperature, max. temperature & propolis content. H. 3D PDP of max. temperature, organic carbon & propolis content. I. 3D PDP of phosphorus, min. temperature & propolis content. J.3D PDP of phosphorus, max. temperature & propolis content. K. 3D PDP of phosphorus, organic carbon & propolis content.

Partial dependence plots with two variables show the mutual contribution of the variables to the response. Such plots help identify the optimum range of predictor values for a maximum value of the response. Two variable PDPs Figs 9A–9K and 10A–10K are generated for the top five important variables (altitude, phosphorous content, maximum average temperature, minimum average temperature & soil organic carbon content) to understand the mutual influence of these variables on response. For each pair of predictors, two PDPs are generated; a 2D contour plot & 3D partial dependence plot. In each plot, the color scale on the right-hand side shows the color as a measure of propolis content.

From these plots, it is evident that lower value of altitude i.e. 0 to 200m approx. (Figs 9B–9E and 10B–10E) and a soil phosphorous content between 50 to 175kg/ha hasa favourable effect on propolis content **(**Figs 9E, 9I–9K, 10E and 10I–10K). Similarly, lower values of maximum & minimum average temperature i.e. below 33°C& 17°C were found to increase propolis content. For organic carbon, two ranges exist (as evident from a single variable PDP for organic carbon and two variable PDPs for organic carbon, maximum average temperature, Figs 9F and 10F & organic carbon-altitude, Figs 9D and 10D) which favor higher propolis content. The approximate ranges are between 5 to 9 kg/ha and below 2kg/ha.

## Discussion

A data distribution study is necessary before implementing a machine learning model as machine learning models are also influenced by the data distribution of predictors. In this data set, not all variables are normally distributed. In such cases, model selection plays a crucial role. Some models need further data processing. However, tree-based models & artificial neural networks can also perform well when the data distribution is not normal [32,33].

A correlation study among the predictors has significance in model evaluation. When Pearson’s correlation coefficient value is between 0 to 0.1, 0.1 to 0.39, and 0.4 to 0.69, the correlation is negligible, weak & moderate respectively. Correlation is strong when Pearson’s correlation coefficient value is between 0.7 and 0.89 & very strong when the correlation coefficient value is between 0.9 and 1 [34]. A correlation study among the predictors has significance in model evaluation. The machine learning algorithms are affected if a correlation exists among the predictors [35].

The exploratory analysis shows the correlations among the predictors are within a range ofnegligible to strong. Hence, the model’s performance may get influenced due to such multicollinearity among the variables. In such cases, tree based models, and artificial neural networks perform well & are not influenced by multicollinearity among the predictors.

From the comparative study of eight different machine learning models, it is evidentthat the artificial neural network (ANN) model has outperformed all other models with the lowest MAE & RMSE values & highest value of the R-squared (Table 3 and Fig 3).

The use of artificial neural networks (ANN) is suggested as a promising way of predicting propolis oil content. This technology not only gives new possible approaches for bio-compound study into other plants and other environmental conditions, but it also provides new potential approaches for bio-compound research into other plants and other environmental situations. ANN has been proposed by many academics as a predictive method for optimizing operating parameters during the extraction of diverse natural products [36–44].

Akbar et al. (2018) reported on the use of ANNmodeling for the optimization and prediction of essential oil yield in turmeric *(Curcuma longa L*.*)*. By analyzing the soil and environmental conditions in the eight agro-climatic areas of Odisha and using data on the essential oil of 131 turmeric germplasms, they created a model. The ANN model was trained and tested on each sample with 11 parameters. With an R2 value of 0.88, the results demonstrated that multilayer-feedback neural networks with 12 nodes (MLFN-12) were the most appropriate and logical model to utilizeThis study shows that an ANN-based prediction model is a good method for forecasting oil output at a new location and for optimizing turmeric oil yield at a specific site by adjusting the prediction model’s changeable parameters, and as a result, it has enough commercial relevance.Niazian et al. (2018) used artificial neural networks (ANN) and multiple regression models (MLR) to forecast the oil content of ajowan based on easily quantifiable plant characteristics. Four characteristics (number of rays, number of pedicels, number of flowers per umbellet, and number of umbellets in an umbel) were chosen as input variables in both artificial neural network and multiple linear regression models by simple correlation analysis. Using the SigmoidAxon transfer function and two hidden layers of an artificial neural network, the essential oil concentration of ajowan was accurately predicted with a root mean square error (RMSE) of 0.192%, a mean absolute error (MAE) of 0.112%, and a determination coefficient (R2) of 0.901.ANN outperformed MLR in terms of performance, with an RMSE of 0.262 and an R2 of 0.748. Based on stepwise regression and ANN analyses the most important features for the oil content of ajowan were the number of umbellets in an umbel and thenumber of flowers per umbellet and these qualities can be given as selection criteria for the essential oil content of ajowan.B. monnieri wild accessions were gathered from 81 sites in various eastern Indian areas (Odisha and West Bengal) to create an experimental dataset. According to the ANN results, a single hidden layer with 11 neurons, or the 13-11-1 structure of a multilayer perceptron (MLP) neural network, had the best ability to estimate the amount of bacoside A in a sample. With a coefficient of determination (R 2), a root mean square error (RMSE), and a mean absolute percentage error (MAPE) of 0.90, 0.16, and 7.76%, respectively, the constructed ANN model demonstrated a stronger predictive capacity for the training dataset.Additionally, the findings of the sensitivity analysis revealed that nitrogen concentrations and altitude had the greatest effects on the content of bacoside A. When evaluated at a new location, the ANN model showed a prediction accuracy of 93.60% for the presence of bacoside A. According to the study’s findings, bacoside A content in *B*.*monnieri (L*.*)* at a certain area can be predicted and optimized using an ANN model.

The advantage of ANN over other statistical modeling techniques is that it does not require the inference of a prior data structure and may detect nonlinear correlations and complicated interactions, exposing previously unknown linkages between input parameters [45]. The performance of the ANN model was assessed using root mean square error (RMSE), coefficient of determination (R^2^), mean absolute error (MAE), symmetric mean absolute percentage error (MAPE) & NSE coefficient. Root means square error (RMSE) was used as a performance measure to select the best model. Though the data set has multicollinearity among variables, the RMSE can be used as a performance measure [46]. The developed model performed well when evaluated with the above-mentioned performance measures. The linearity test & slope intercept test were also added to provide the model’s credibility. A similar study with a high predictive analysis of the ANN model was previously published, with the error values of the ANN model being lower [47–49].

ANNs do, however, have several important disadvantages. The usage of pricey graphics processing units (GPUs) that provide parallel processing is typically required for ANNs. Sharing an ANN after training is challenging. ANNs tend to overfit the training set. Because the size and structure of an ANN are mostly determined by trial and error, overfitting is likely due to this factor among other others.Experience and trial and error are frequently used to determine the size and structure of ANNs. Convergence on a prediction or solution is not guaranteed by ANNs. An ANN can accurately approximate a target function by selecting the appropriate parameters, or "hyperparameters" in ANN parlance. Achievable solutions like this don’t always exist, though. ANNs require a considerable amount of training time.

The correlation strengths among variables are also represented by partial dependence plots. Most of the correlation among variables is weak to moderate. Minimum average temperature & minimum average relative humidity have a strong correlation. In such cases, ANN models provide promising results and are free from any biases due to multicollinearity [50]. So, PDPs generated through artificial neural network models are suitable to study the change in response to predictors.

The coefficients in a generalized linear model are partially equivalent to those in an ANN, the weights that connect neurons. The weights’ cumulative influence on model predictions reflects the relative importance of predictors in their relationships with the outcome variable. In an ANN,there are numerous weights connecting one predictor to the outcome. An ANN’s high number of adjustable weights makes it incredibly flexible in modeling nonlinear phenomena, but it also makes interpretation difficult. The relative importance of a predictor, according to Garson, may be assessed by examining the model weights [51,52]. There are no linkages between each predictor of interest and the outcome. When all weights about a predictor are combined and scaled, a single value ranging from 0 to 1 is generated that shows relative predictor relevance. The Neural Networking Tools (version 1.5.1) package in R can be used to calculate relative relevance [31].

Friedman, 2001 proposed partial dependence plots (PDP) to show how one or two variables affect the model’s predictions. We also propose a new display that, like a scatter plot matrix, shows all pairwise partial dependence plots in a matrix-style layout with a univariate partial dependence plot on the diagonal. With this presentation, the analyst can observe at a glance how important pairs of variables affect the fit. Interpretation is aided once more by careful sequencing of the variables [53].

This is the first study to look at the impact of environmental and soil nutritional parameters on propolis oil content in 10 different Odisha agro-climatic areas. A combination of two or more factors has a greater impact on propolis oil content than a single element, according to the study. The current study found that environmental variables, such as soil macronutrients, can influence propolis oil content. The current study discovered that adjusting height, maximum average temperature, and phosphorus content in the soil can maximize propolis oil content in the ANN model. Differences in Propolis oil content are influenced by the parameters mentioned above, which need to be investigated further. The ANN model was used to predict the amount of propolis oil in a new location.

## Conclusions

Artificial Neural Network (ANN) models were constructed in propolis for the prediction and optimization of oil yield in this study. The multilayer feed-forward neural network model was determined to be the most efficient for oil yield optimization, with a coefficient of determination value of 0.93. The oil content of propolis can also be enhanced using the ANN model by adjusting the model’s input parameters (altitude, phosphorus, and maximum average temperature). The ANN model created in this work could potentially be utilized to forecast propolis oil yield at a new location. Experimental findings backed up the forecast. The created ANN model could be useful in the manufacture of high-oil-content propolis and hence has commercial value.

## Supporting information

S1 Table Climatic data for propolis from different Agro-climatic regions of Odisha. (DOCX)

S2 Table Physicochemical properties of soil samples collected from different Agro-climatic regions of Odisha. (DOCX)
