# Supplementary material for: Developing a computational toolbased on an artificial neural network for predicting and optimizing propolis oil, an important natural product for drug discovery
Source: PLoS One. 2023 May 8;18(5):e0283766. doi: 10.1371/journal.pone.0283766 (PMC10166476; doi:10.1371/journal.pone.0283766)
Supplement: S2 Table — (DOCX) [file pone.0283766.s002.docx]

**SUPLIMENTARY FILE**

**S2 Table: Physicochemical properties of soil samples collected from different Agro-climatic regions of Odisha**

| SL. No. | Agroclimatic Zones | Districts | Accession no. | Organic Carbon (%) | Nitrogen | Phosphorous | Potassium |
| --- | --- | --- | --- | --- | --- | --- | --- |
| 1. | East & South East Coastal Plain | Jagatsingpur | P1 | 1.59 | 236.2 | 79.4 | 542.2 |
|  |  |  | P2 | 1.61 | 235.9 | 81.4 | 538.7 |
|  |  | Khurda | P3 | 1.11 | 503.7 | 161.7 | 918.4 |
|  |  |  | P4 | 1.41 | 166.5 | 217.2 | 493.2 |
|  |  | Puri | P5 | 0.99 | 305.31 | 265.1 | 796.94 |
|  |  |  | P6 | 1.7 | 270.4 | 63.4 | 408.3 |
|  |  | Nayagarh | P7 | 0.76 | 250 | 169.05 | 491.9 |
|  |  |  | P8 | 0.83 | 250.4 | 169.02 | 491.2 |
| 2. | North Eastern Coastal Plain | Bhadrak | P9 | 0.87 | 352.5 | 64.7 | 201.6 |
|  |  |  | P10 | 0.82 | 352.1 | 64.2 | 201.2 |
|  |  | Balasore | P11 | 3.2 | 340.2 | 83.2 | 602.3 |
|  |  |  | P12 | 3.4 | 179.5 | 281.9 | 209.4 |
|  |  | Jajpur | P13 | 1.8 | 375.4 | 132.2 | 302.5 |
|  |  |  | P14 | 2 | 381.5 | 130.3 | 294.6 |
| 3. | North Eastern Ghat | Ganjam | P15 | 3.1 | 183.7 | 280.3 | 208.3 |
|  |  |  | P16 | 3.4 | 340.6 | 83.8 | 602.4 |
|  |  | Gajapati | P17 | 1.36 | 166.2 | 217.5 | 493.9 |
|  |  |  | P18 | 1.27 | 176 | 26.58 | 78.1 |
|  |  | Kandhamal | P19 | 0.52 | 141.2 | 37.2 | 519 |
|  |  |  | P20 | 0.58 | 141.3 | 37.5 | 519.7 |
| 4. | Mid Central Table Land | Angul | P21 | 0.94 | 162.3 | 33.2 | 771.6 |
|  |  |  | P22 | 0.91 | 162.7 | 33.7 | 771.8 |
|  |  | Dhenkanal | P23 | 1.79 | 562.5 | 132.4 | 921.6 |
|  |  |  | P24 | 1.81 | 562.1 | 132.1 | 921.3 |
|  |  | Cuttack | P25 | 1.5 | 251.2 | 96.3 | 306 |
|  |  |  | P26 | 3.61 | 152.4 | 72.6 | 89.1 |
| 5. | Western Central Table Land | Boudh | P27 | 0.32 | 125 | 127.91 | 309.12 |
|  |  |  | P28 | 0.35 | 123 | 128.1 | 310.1 |
|  |  | Bargarh | P29 | 5.2 | 140.3 | 142.3 | 30 |
|  |  |  | P30 | 5.4 | 140.7 | 141.9 | 32 |
|  |  | Jharsuguda | P31 | 0.94 | 112.5 | 75.5 | 603.46 |
|  |  |  | P32 | 0.91 | 112.4 | 74.9 | 602.9 |
| 6. | Eastern Ghat High Land | Nawarangpur | P33 | 1.14 | 175 | 26.48 | 77.95 |
|  |  |  | P34 | 1.01 | 113 | 75.3 | 603.8 |
|  |  | Rayagada | P35 | 3.27 | 316.2 | 29.3 | 924.8 |
|  |  |  | P36 | 3.98 | 164.5 | 82.4 | 93.4 |
|  |  | Koraput (East) | P37 | 4.61 | 381.2 | 32.4 | 961.3 |
|  |  |  | P38 | 4.74 | 380.6 | 32.1 | 960.8 |
| 7. | North Central Plateau | Mayurbhanj (South) | P39 | 2.23 | 175 | 50.72 | 73.92 |
|  |  |  | P40 | 4.01 | 164.3 | 82.6 | 93.1 |
|  |  | Keonjhar (North) | P41 | 3.57 | 152.7 | 72.8 | 89.3 |
|  |  |  | P42 | 3.65 | 152.5 | 72.4 | 89.1 |
|  |  | Mayurbhanj (North) | P43 | 3.98 | 164.5 | 82.4 | 93.4 |
|  |  |  | P44 | 4.72 | 383.2 | 32.7 | 959.6 |
| 8. | South Eastern Ghat | Keonjhar (South) | P45 | 8.4 | 261 | 145.2 | 391.2 |
|  |  |  | P46 | 8 | 231 | 152 | 308.7 |
|  |  | Koraput (South-East) | P47 | 2.3 | 242.3 | 132.7 | 296.3 |
|  |  |  | P48 | 3.4 | 340.4 | 83.5 | 602.4 |
|  |  | Malkangiri | P49 | 6.36 | 216.4 | 121.2 | 386.4 |
|  |  |  | P50 | 5.1 | 140.1 | 142.5 | 29.8 |
| 9. | North Western Plateau | Sundargarh | P51 | 1.06 | 285.3 | 161.2 | 603.4 |
|  |  |  | P52 | 1.1 | 285.1 | 161.4 | 603.1 |
|  |  | Deogarh | P53 | 8.1 | 230.4 | 152.3 | 308.5 |
|  |  |  | P54 | 8.3 | 230.5 | 152.7 | 308.8 |
|  |  | Sambalpur | P55 | 8.6 | 262.5 | 139.75 | 594.8 |
|  |  |  | P56 | 8.9 | 263.7 | 139.65 | 592.6 |
| 10. | Western Undulating Zone | Kalahandi | P57 | 2.4 | 240.3 | 76.3 | 813.7 |
|  |  |  | P58 | 2.5 | 240.4 | 76.2 | 813.6 |
|  |  | Bolangir | P59 | 8.9 | 290.4 | 89.1 | 503.1 |
|  |  |  | P60 | 8 | 230.2 | 152.7 | 308.3 |
|  |  | Nuapada | P61 | 1.6 | 270.3 | 63.2 | 408.1 |
|  |  |  | P62 | 1.18 | 175.4 | 26.51 | 77.81 |
